# Quantitative Proteomics for the Identification of Differentially Expressed Proteins in the Extracellular Vesicles of Cervical Cancer Cells

**DOI:** 10.3390/v15030702

**Published:** 2023-03-08

**Authors:** Víctor Acevedo-Sánchez, Roy S. Martínez-Ruiz, Sergio R. Aguilar-Ruíz, Honorio Torres-Aguilar, Pedro Chávez-Olmos, Efraín Garrido, Rafael Baltiérrez-Hoyos, María de los A. Romero-Tlalolini

**Affiliations:** 1 Facultad de Medicina y Cirugía, Universidad Autónoma Benito Juárez de Oaxaca, Ex Hacienda de Aguilera S/N, Calz. San Felipe del Agua, Oaxaca de Juárez 68120, Mexico; 2 Facultad de Ciencias Químicas, Universidad Autónoma Benito Juárez de Oaxaca, Av. Universidad S/N, Cinco Señores, Oaxaca de Juárez 68120, Mexico; 3 Departamento de Genética y Biología Molecular, Centro de Investigación y de Estudios Avanzados del Instituto Politécnico Nacional, Av. Instituto Politécnico Nacional 2508, Col. San Pedro Zacatenco, Delegación Gustavo A. Madero, Mexico City 07360, Mexico; 4 Facultad de Medicina y Cirugía, CONACYT—Universidad Autónoma Benito Juárez de Oaxaca, Ex Hacienda de Aguilera S/N, Calz. San Felipe del Agua, Oaxaca de Juárez 68120, Mexico

**Keywords:** extracellular vesicles, exosomes, proteomics, HPV, HeLa cells, cancer

## Abstract

The extracellular vesicles (EVs) in a tumoral microenvironment can exert different functions by transferring their content, which has been poorly described in cervical cancer. Here, we tried to clarify the proteomic content of these EVs, comparing those derived from cancerous HPV (+) keratinocytes (HeLa) versus those derived from normal HPV (–) keratinocytes (HaCaT). We performed a quantitative proteomic analysis, using LC-MS/MS, of the EVs from HeLa and HaCaT cell lines. The up- and downregulated proteins in the EVs from the HeLa cell line were established, along with the cellular component, molecular function, biological processes, and signaling pathways in which they participate. The biological processes with the highest number of upregulated proteins are cell adhesion, proteolysis, lipid metabolic process, and immune system processes. Interestingly, three of the top five signaling pathways with more up- and downregulated proteins are part of the immune response. Due to their content, we can infer that EVs can have a significant role in migration, invasion, metastasis, and the activation or suppression of immune system cells in cancer.

## 1. Introduction

Cervical cancer is the fourth leading cause of cancer death in women and the fourth with the highest incidence, with 341,831 deaths and 604,127 new cases worldwide reported in 2020 [1]. Persistent infection with high-risk human papillomaviruses (hr-HPVs) contributes to the development of cervical cancer. Epidemiological studies have identified hr-HPV presence in up to 99.7% of cervical cancer cases [2]. Despite the oncogenic capacity of these viruses, HPV infection alone is insufficient for the development of cervical cancer, as only a tiny fraction of women infected with HPV develop cervical cancer [3], indicating that multiple factors are required for progression [4]. Among these several factors is the tumor microenvironment (TME), which is not only a consequence of cancer progression but also contributes to its development [3]. In addition to tumor cells, the TME comprises immune system cells, stromal cells, blood, lymphatic vessels, the extracellular matrix, and secreted molecules, which impact immunosuppression, chemoresistance, cancer progression, and metastasis [5]. Tumor cells interact with the microenvironment through cell–cell interactions, releasing various growth factors, cytokines, chemokines, and enzymes. Tumors need to be considered a multidimensional spatiotemporal unit where an additional communication mechanism between cells in the TME is mediated through extracellular vesicles (EVs) [6,7]. Extracellular vesicles are particles naturally released by cells, delimited by a lipid bilayer. They are classified into three types according to their biogenesis: exosomes, microvesicles or ectosomes, and apoptotic bodies [8,9]. Exosomes are particles of approximately 30–100 nm in size with an endosomal origin and are released through the fusion of multivesicular bodies with the plasma membrane [10]. Microvesicles (MVs) are generated from direct budding from the cytoplasmic membrane and range in size from 100 to 1000 nm. Apoptotic bodies are larger particles with a diameter of 1–5 μm released by apoptotic cells [8]. EVs contain nucleic acids, lipids, and proteins.

The transfer of this content can promote the reprogramming of functions in the recipient cell [11]. Proteins are one of the main components of EVs; they can be present within the EV lumen or on its membrane, and protein packing and sorting in EVs are regulated [12]. Proteomic studies have shown that EVs contain proteins common among all EVs, regardless of the cell type from which they originate, while another percentage of proteins are cell-specific, reflecting the cell type and physiological or pathological conditions [11]. Much of this information is compiled in Vesiclepedia, a database of proteins, RNA, and lipids identified in all EV types [13]. Despite the increasing number of publications about the role of EVs in cervical cancer, information on the protein content of these EVs is still limited [14]. The protein content is of great importance because proteins are the effector molecules of various processes in the cell, and it has been shown that proteins transported in EVs maintain their functional activity when taken up by recipient cells [15]. The use of quantitative proteomic techniques has allowed the characterization and determination of the number and quantity of proteins in EVs in various pathological conditions, contributing to the understanding of the mechanisms of their biogenesis and the functions of EVs in such conditions [16]. In cervical cancer EVs, only the identification of some proteins has been described, such as those of the apoptosis inhibitor family [17], some proteins related to oxidative stress [18], and Hedgehog signaling [19]. Additionally, Lin et al. suggested that HeLa-cell-derived exosomes elicit endoplasmic reticulum stress in endothelial cells, and their protein content could mediate this effect; therefore, they performed LC-MS/MS, but only 44 proteins were identified [6]. So, the total content of proteins in the EVs of cervical cancer cells and their levels compared with other keratinocytes remain unknown.

In this study, we characterized the entire protein content of the EVs derived from cervical cancer cells (HeLa cell line) and the EVs isolated from non-tumorigenic human keratinocytes (HaCaT cell line) through a label-free based quantitative proteomic analysis as a first step to determine the main protein in EVs with a potential role in cervical cancer. We identified the differentially expressed proteins between the EV group secreted by cervical cancer cells (HeLa) and non-tumorigenic keratinocytes (HaCaT). In addition, we identified the cellular component, molecular function, biological processes, and biological pathways in which the differentially expressed proteins are present through bioinformatic analysis.

## 2. Materials and Methods

### 2.1. Cell Cultures

The human cervical cancer cell line HeLa was purchased from the American Type Culture Collection (CCL-2, ATCC). The spontaneously immortalized human keratinocyte cell line HaCaT, used as non-tumorigenic keratinocytes, was donated by Ph.D. Efrain Garrido along with C-33 A. HeLa and HaCaT cell lines were cultured in Dulbecco’s modified Eagle’s medium (DMEM) low glucose (L0066, Biowest, Nuaillé, France) and C-33 A in DMEM-F12 (L0092, Biowest), all supplemented with 10% fetal bovine serum (FBS) (S1650, Biowest) and penicillin–streptomycin (L0022, Biowest) 100 U/mL. The cell lines were incubated at 37 °C in a humidified atmosphere with 5% CO_2_. The HPV status in both cell lines was evaluated using PCR with the universal oligonucleotides MY09/MY11 and was only detected in HeLa (Supplementary Figure S1).

### 2.2. Extracellular Vesicle (EV) Isolation

EVs were obtained from 1 × 10^6^ cells and cultured in 100 mm dishes until 80–90% confluence. Then, the culture medium was removed, the cells were washed twice with 1X PBS, and a fresh culture medium without FBS was added. The cell cultures were incubated at 37 °C and 5% CO_2_ for 24 h. Subsequently, the cell culture medium was collected and centrifuged at 650× *g* for 5 min at room temperature to remove the cells. The pellets obtained were discarded, and the supernatants were used to isolate the extracellular vesicles using a miRCURY Exosome Kit (76743, Qiagen, Hilden, Germany) according to the manufacturer’s instructions. Briefly, the supernatants were centrifuged at 3200× *g* for 15 min at room temperature to remove cell debris and apoptotic bodies. The supernatants were transferred to new tubes, where precipitation buffer B was added (400 μL of precipitation buffer B per mL of cell culture medium), and the samples were incubated at 4 °C overnight. After incubation, all the samples were centrifuged at 10,000× *g* for 30 min at 20 °C, and the supernatants were removed. The extracellular vesicles were resuspended in a RIPA buffer for protein extraction or in 2% glutaraldehyde for identification via transmission electron microscopy.

### 2.3. Extracellular Vesicle Identification

#### 2.3.1. Transmission Electron Microscopy (TEM)

First, 10 µL of the EV fixed with 2% glutaraldehyde (16200, Electron Microscopy Sciences, Hatfield, PA, USA) was deposited onto a carbon-coated copper grid (400-mesh) for 2 min. The excess sample was decanted onto filter paper, and the samples in grids were negatively stained with 10 μL of 2% uranyl acetate solution for 3 min. Excess dye was decanted onto filter paper, and the samples were allowed to dry at room temperature in the dark. Imaging was performed at an acceleration voltage of 80 kV with a JEM 1400 transmission electron microscope (JEOL, Tokyo, Japan). At least five fields were documented for each kind of EV.

#### 2.3.2. Western Blot

Equivalent amounts of total protein from extracellular vesicles and cells were separated using SDS–PAGE and transferred to a polyvinylidene fluoride membrane (88520, Thermo Fisher Scientific, Waltham, MA, USA). The membrane was blocked with 5% non-fat milk in 1X TBS-T (0.05%) for 2 h at room temperature and subsequently incubated with primary antibodies anti-HSP90 α/β (1:3000, sc-13119, Santa Cruz Biotechnology, Dallas, TX, USA), anti-HSP70 (1:1000, sc-24, Santa Cruz Biotechnology), anti-CD9 (1:250, sc-13118, Santa Cruz Biotechnology), anti-Calnexin (1:400, sc-23954, Santa Cruz Biotechnology), anti-vitronectin (1:500, sc-74484, Santa Cruz Biotechnology), and anti-β-catenin (1:500, ab2365, Abcam, Cambridge, UK) overnight at 4 °C. The membranes were washed with TBS-T and incubated with a secondary antibody (1:5000, 115-035-003, Jackson ImmunoResearch Laboratories, West Grove, PA, USA, or 1:3000, 65-6120, Invitrogen, Waltham, MA, USA) for 2 h at room temperature. The membranes were washed with TBS-T three times and scanned on a C-DiGit Blot scanner (LI-COR Biosciences, Lincoln, NE, USA) using an Immobilon Crescendo Western HRP Substrate (WBLUR0100, Millipore, Burlington, MA, USA). Image Studio Digits v.5.2 software (LI-COR Biosciences, Nebraska, USA) was used for image acquisition.

### 2.4. Protein Extraction and Quantification

Extracellular vesicles were lysed with a radioimmunoprecipitation assay (RIPA) buffer (sc-24948A, Santa Cruz Biotechnology), and protein concentration was determined using a Pierce BCA Protein Assay Kit (23225, Thermo Fisher Scientific) according to the manufacturer’s instructions.

### 2.5. Sodium Dodecyl Sulfate Polyacrylamide Gel Electrophoresis (SDS–PAGE)

Briefly, 20 µg of protein from extracellular vesicles was mixed with a 4X Laemmli buffer (250 mM Tris-HCl pH = 6.8, 40% glycerol, 4% SDS, 10% β-mercaptoethanol, and 0.02% bromophenol blue) and boiled at 96 °C for 5 min. The samples were loaded into each lane of the gel, and protein separation was performed using 10% SDS–PAGE at 25 mAmp for 30 min. After electrophoresis, the gel was stained with Coomassie brilliant blue G-250 (20279, Thermo Fisher Scientific) for visualization, and the blue-stained gel fragments were cut and washed twice by shaking with MilliQ water for 5 min for label-free quantitative proteomic analysis.

### 2.6. Label-Free Based Quantitative Proteomic Analysis

#### 2.6.1. In-Gel Digestion

Each Coomassie blue stained gel slice was cut into cubes of 1 mm^3^ and washed with 50 mM ammonium bicarbonate/acetonitrile (NH_4_HCO_3_/ACN) (1:1, *v*/*v*) for 30 min, and the supernatant was discarded. Then, 500 µL of ACN (Sigma-Aldrich, Darmstadt, Germany) was added, and after incubation for 30 min, the ACN was removed. The proteins were reduced with 10 mM DTT (Sigma-Aldrich) for 1 h at 56 °C, followed by alkylation with 50 mM IAA (Sigma-Aldrich) for 30 min at room temperature in the dark. For the enzymatic hydrolysis of proteins, trypsin (0.01 μg/μL, Promega, Fitchburg, WI, USA) was added to a 50 mM NH_4_HCO_3_ solution and incubated on ice for 45 min. The digestion solution was removed, and 20 μL of 50 mM NH_4_HCO_3_ was added. The supernatants were recovered in a new tube, and extraction was repeated with 50 mM NH_4_HCO_3_/ACN solution (1:2, *v*/*v*). Then, the solution was recovered and mixed with the supernatant of the first extraction. Finally, the extracted peptides were lyophilized and resuspended in 20 μL of 0.1% formic acid (Sigma-Aldrich) before LC-MS/MS analysis.

#### 2.6.2. Nano LC-MS/MS Analysis

The peptides from protein digestion were analyzed via LC-MS/MS using an Ultimate 3000 nano UHPLC system coupled with a Q Exactive HF mass spectrometer (Thermo Fisher Scientific, USA) with an ESI nanospray source. Briefly, 1 µg of the sample was loaded onto a trapping column (PepMap C18, 100 Å, 100 μm × 2 cm, 5 μm, Thermo Fisher Scientific, USA) and washed with 0.1% formic acid in water (buffer A). The trapped mixture was then eluted on an analytical column (PepMap C18, 100 Å, 75 μm × 50 cm, 2 μm, Thermo Fisher Scientific, USA) through a linear gradient from 2–8% buffer B (0.1% formic acid in 80% acetonitrile) for 3 min, 8–20% buffer B for 56 min, 20–40% buffer B for 37 min, and then 40–90% buffer B for 4 min at a flow rate of 250 nL/min. Full mass spectra were acquired in the range of 300–1650 m/z at a resolution of 60,000 at 200 m/z, and the automatic gain control target for the full scan was set to 3 × 10^6^. The MS/MS scan was operated in a Top 20 mode using the following settings: resolution 15,000 at 200 m/z; automatic gain control target 1 × 10^5^; maximum injection time 19 ms; normalized collision energy at 28%; isolation window of 1.4 Th; charge state exclusion: unassigned, 1, >6; and dynamic exclusion for 30 s. A total of four samples were analyzed, each group in duplicate, i.e., two samples of the extracellular vesicles from HeLa cells and two samples of the extracellular vesicles from HaCaT cells.

#### 2.6.3. Analysis of LC-MS/MS Data

The raw MS files were analyzed and searched against human and human papillomavirus type 18 protein database (UniProt) based on the species of the samples using MaxQuant software (v1.6.1.14). The parameters were set as follows: protein modifications were carbamidomethylation (C) (fixed) and oxidation (M) (variable); the enzyme specificity was set to trypsin; the maximum missed cleavages were set to 2; the precursor ion mass tolerance was set to 10 ppm, and MS/MS tolerance was 0.6 Da. The data obtained after processing in MaxQuant software were analyzed using Perseus software (v1.6.15.0). Protein abundance for each one of the samples was determined based on the LFQ intensity data. First, the filtering of the data was performed to remove the proteins only identified by site, reverse matches, and potential contaminants. Subsequently, the LFQ intensities were Log2-transformed to obtain a normal data distribution. Then, sample grouping was performed (first group: HeLa EVs; second group: HaCaT EVs), and valid values were filtered according to the groups (two valid values in at least one group). We then imputed missing values from the normal distribution by selecting a downward shift of 1.8 and a width of 0.3 standard deviations. Differentially expressed proteins were determined using a two-sample Student’s *t*-test. A fold change (FC) = 2 and a permutation-based FDR of 0.05 were used.

### 2.7. Bioinformatic Analysis

A comparison of the proteins identified through LC-MS/MS analysis with the Vesiclepedia extracellular vesicle database (accessed 18 September 2022) was performed using FunRich software (v3.1.3). The list of the top 100 proteins reported in EVs was obtained from the Vesiclepedia database at the following link: http://microvesicles.org/extracellular_vesicle_markers. The gene ontology analysis of the differentially expressed proteins was performed through the gene annotation co-occurrence discovery (GeneCodis4). The participation of the differentially expressed proteins in biological pathways was analyzed using the Reactome pathway database. GO (CC, MF, and BP) and biological pathway categories with a *p*-value < 0.05 were considered statistically significant. The protein–protein interaction (PPI) network was constructed from the differentially expressed proteins using the Search Tool for the Retrieval of Interacting Genes/Proteins (STRING) v11.5 (http://string-db.org/. Accessed 20 December 2022) database. To generate the PPI network, the species “*Homo sapiens*” was selected; the type of network was a full STRING network, with a required score of high confidence (0.700) and an FDR stringency of 5% (medium).

## 3. Results

### 3.1. Identification of Extracellular Vesicles

**Figure 1:**
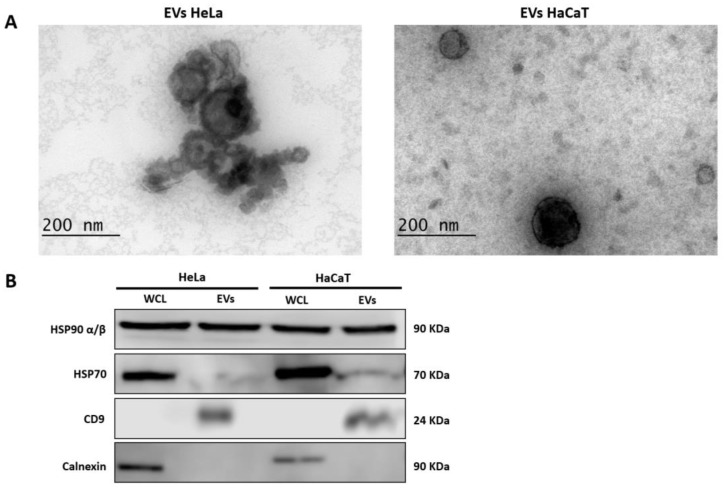
Characterization of EVs derived from HeLa and HaCaT cells: (**A**) visualization of EVs using TEM (×200,000); (**B**) Western blot analysis of EV markers HSP90 α/β, HSP70, and CD9. Calnexin protein was used as a marker of EV purity. EVs: extracellular vesicles; WCL: whole-cell lysate.

To determine the protein content of the EVs derived from cervical cancer cells, we first isolated the EVs from the supernatant of HeLa (cervical cancer cells) and HaCaT (control cells) cell cultures and studied their characterization using transmission electron microscopy (TEM) and Western blot. As observed in the results of TEM analysis, the isolated EVs showed a spheroidal or cup-shaped morphology and a diameter of less than 200 nm (Figure 1A). Next, we identified EV marker proteins HSP90 α/β, HSP70, and CD9. Western blot analysis identified all three proteins in the EV lysates from both cell lines (Figure 1B). In contrast, the presence of calnexin protein, commonly employed to assess EV purity, was only identified in whole-cell lysates (WCL) (Figure 1B), which indicates that the isolated EVs were not contaminated by cellular debris. These results show that the isolation of EVs (enriched in exosomes and subsequently referred to as EVs) was successful and with adequate purity for subsequent proteomic analysis.

### 3.2. Analysis of Protein Content in HeLa and HaCaT EVs and Comparison with the Vesiclepedia Database

**Figure 2:**
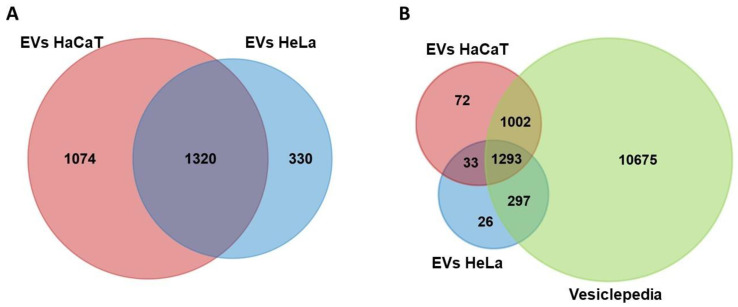
Venn diagrams of proteins identified in EVs: (**A**) comparison of proteins identified in EVs from HeLa and HaCaT cells; (**B**) comparison of proteins in EVs from HeLa and HaCaT cells included in the Vesiclepedia database.

For the characterization of the protein content of the EVs from HeLa and HaCaT cells, 20 µg of protein and two biological replicates of each kind of vesicle were analyzed. Using trypsin, proteins were enzymatically digested into peptides, which were then examined via liquid chromatography and tandem mass spectrometry (LC-MS/MS). The raw MS files were analyzed and searched in the UniProt human protein database using the MaxQuant software. A total of 2724 proteins were identified, 1650 of which were found in HeLa EVs and 2394 in HaCaT EVs (Supplementary Table S1). Both EVs had 1320 proteins in common (Figure 2A). The mass spectrometry data from HeLa EVs were also analyzed in the HPV type 18 protein database; however, the presence of viral proteins was not identified. The proteins of the EVs from HeLa and HaCaT cells were analyzed with the FunRich program to identify the proteins not previously reported in the Vesiclepedia database. We found that a high percentage of proteins from the EVs of HeLa (96.42%) and HaCaT (95.61%) cells had been previously reported in this database. However, a total of 131 proteins were not in Vesiclepedia, 59 of HeLa’s EVs and 105 of HaCaT’s EVs (Figure 2B and Supplementary Table S2), indicating that they are unique proteins to this type of EV.

In addition, a comparison of the proteins identified in our mass spectrometry analysis with that of the top 100 proteins reported in the Vesiclepedia database, which includes the primary markers for EVs, was performed. In total, 87 proteins of this top 100 were identified in our analysis, and even better, the 20 most frequently reported proteins (top 20) were identified in both replicates of the EVs from each cell line. The average abundance values of the proteins included in the top 100, as well as those of the top 20, were determined based on the normalized label-free quantitation (LFQ) intensity, and they are displayed in Table 1 and Table S3. Identifying these proteins, including the specific markers for EVs, provided proteomic support for the isolation of our EVs.

### 3.3. Differential Expression of Proteins Identified in the EVs

**Figure 3:**
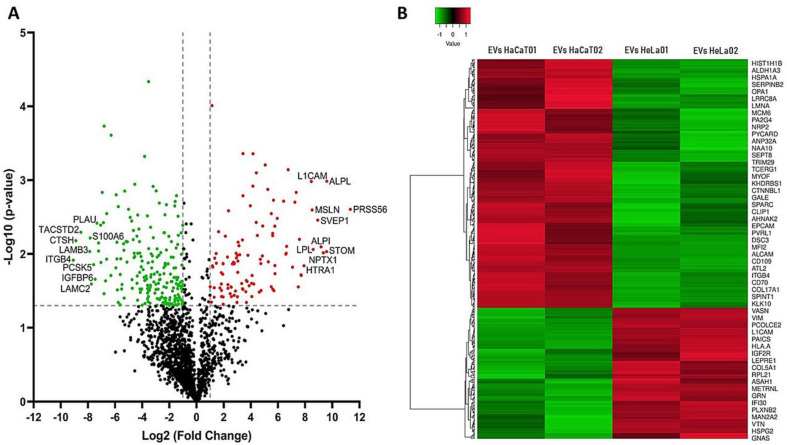
Differentially expressed proteins in EVs: (**A**) volcano plot shows the differentially expressed proteins between cervical cancer EVs (HeLa’s EVs) and control (HaCaT’s EVs). Significantly upregulated proteins (FC ≥ 2) are indicated in red, and significantly downregulated proteins (FC ≤ −2) in green; proteins without significant changes are seen in black. The X-axis shows the level of cervical cancer fold change versus control (base 2 log transformation), and the Y-axis shows the negative logarithm (base 10 log transformation) of the *p*-value; (**B**) hierarchical clustering heat map of differentially expressed (*p*-value < 0.05) upregulated (red) or downregulated (green) proteins in the cervical cancer group compared with those of the control group. HeLa01 and HeLa02 EVs correspond to the two biological replicates of the cervical cancer group; HaCaT01 and HaCaT02 EVs correspond to the two biological replicates of the control group.

To determine the differential expression between the protein group of cervical cancer cells’ EVs (HeLa’s EVs) and the control keratinocytes’ EVs (HaCaT’s EVs), the normalized LFQ intensities were compared to establish the protein expression levels. The proteins were considered significantly different when they met a fold change (FC) ≤ −2 or ≥ 2 and *p*-value < 0.05. Figure 3A shows the volcano scatter plot of up- and downregulated cervical cancer EV proteins. Compared with the control group, 322 differentially expressed proteins were identified in the cervical cancer EV group, 111 of which were upregulated (red), and 211 proteins were downregulated (green) (Figure 3A and Supplementary Table S4). The upregulated proteins with the highest FC were PRSS56 (serine protease 56), ALPL (alkaline phosphatase, tissue-nonspecific isozyme), STOM (stomatin), NPTX1 (neuronal pentraxin-1), and ALPI (intestinal-type alkaline phosphatase). The downregulated proteins with the highest FC were ITGB4 (integrin beta-4), CTSH (pro-cathepsin H), TACSTD2 (tumor-associated calcium signal transducer 2), LAMB3 (laminin subunit beta-3), and S100A6 (protein S100-A6). The differentially expressed proteins were hierarchically clustered and represented in a heat map, as shown in Figure 3B and Figure S2. Hierarchical clustering analysis demonstrated significant differences between the cervical cancer EV protein group and the control group, showing different patterns in each group and high similarity between the data pattern of the biological replicates. We randomly selected two proteins to validate these results. As expected, vitronectin, which showed an FC = 4.6, was found in HeLa’s EVs when analyzed using Western blot. On the other hand, β-catenin was observed using Western blot in HaCaT’s EVs, but it was not observed in HeLa’s EVs, in agreement with the proteomic analysis, where an FC = −4.8 was observed (Supplementary Figure S3).

Additionally, through qualitative proteomic analysis, we evaluated the EVs from the C-33 A cell line of keratinocytes to determine whether overexpressed proteins can be found in other cervical cancer cell lines. Interestingly, 63 (57%) of the upregulated proteins in HeLa’s EVs were identified in the EVs from C-33 A. This qualitative analysis was also performed in HeLa cells’ EVs, and the results were consistent with the quantitative analysis.

A Western blot analysis for vitronectin and β-catenin was performed using C-33 A’s EVs or SiHa’s EVs. We could not observe significant differences between C33-A’s EVs or SiHa’s EVs and HaCaT’s EVs for vitronectin like the differences we observed in HeLa’s EVs. However, the downregulation of β-catenin was consistently observed in the EVs from the cancerous cell lines versus HaCaT’s EVs (Supplementary Figure S3 and Table S5).

### 3.4. Gene Ontology (GO) Analysis of Differentially Expressed Proteins

**Figure 4:**
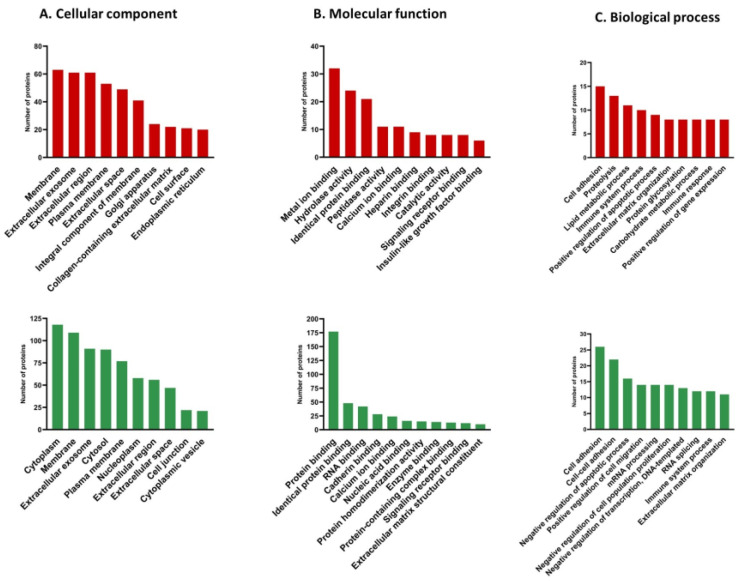
Gene ontology analysis of differentially expressed proteins: (**A**) cellular components, (**B**) molecular functions, and (**C**) biological processes of upregulated (red bars) and downregulated (green bars) proteins from EVs of cervical cancer.

Gene ontology analysis was performed to understand the functions of the 322 differentially expressed proteins in cervical cancer EVs. The proteins were classified according to the cellular component (CC), molecular function (MF), and biological processes (BP) in which they participate (Supplementary Table S6). The analysis was performed using GeneCodis4 (gene annotation co-occurrence discovery), and only statistically significant categories with a *p*-value < 0.05 were considered. Figure 4 shows the ten most enriched terms in the CC, MF, and BP categories.

The upregulated proteins of the EVs from cervical cancer were mainly present in the membrane, extracellular exosome, extracellular region, plasma membrane, and extracellular space. In contrast, the downregulated proteins were associated with the cytoplasm, membrane, extracellular exosome, cytosol, or plasma membrane (Figure 4A).

In the MF category, the upregulated proteins were associated with terms such as metal ion binding, hydrolase activity, identical protein binding, peptidase activity, and calcium ion binding, among others. Protein binding, identical protein binding, RNA binding, cadherin binding, and calcium ion binding were the main terms identified in the downregulated proteins (Figure 4B).

The BPs most associated with the upregulated proteins corresponded to cell adhesion, proteolysis, lipid metabolic process, and immune system process. In contrast, the downregulated proteins were found to participate in cell adhesion, cell–cell adhesion, negative apoptotic process regulation, and positive cell migration regulation (Figure 4C).

### 3.5. Biological Pathway Analyses of Differentially Expressed Proteins

**Figure 5:**
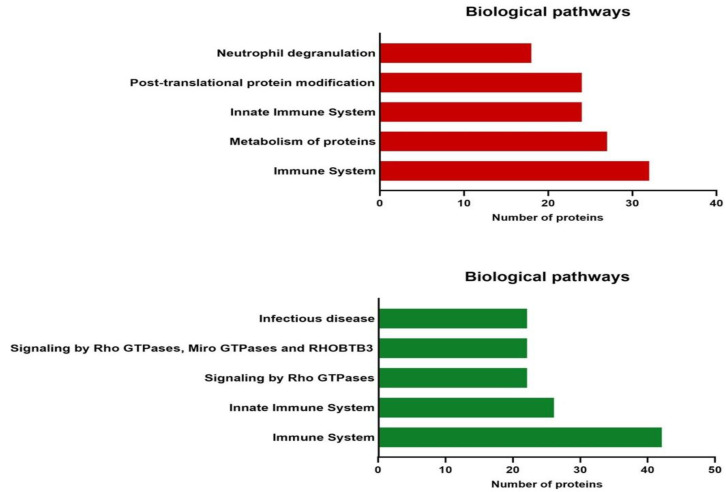
Top five biological pathways involving differentially expressed proteins of cervical cancer EVs. Graphical representation of main Reactome pathways enriched with upregulated (red bars) and downregulated (green bars) proteins with significant *p*-value < 0.05.

We analyzed the Reactome database to obtain more information about the biological pathways in which the differentially expressed proteins of the EVs from cervical cancer participate. Figure 5 depicts the top five pathways with the highest number of differentially expressed proteins with a statistically significant *p*-value (<0.05). This analysis showed 428 signaling pathways for upregulated proteins and 756 for downregulated proteins (Supplementary Table S7). In both biological pathways with up- and downregulated proteins, we found three in the top five related to the immune response: immune system, innate immune system, and neutrophile degranulation. These results suggest that the proteins present in the EVs from cervical cancer could be mainly involved in regulating several immune system pathways and processes.

### 3.6. Interaction Network of Dysregulated Proteins

**Figure 6:**
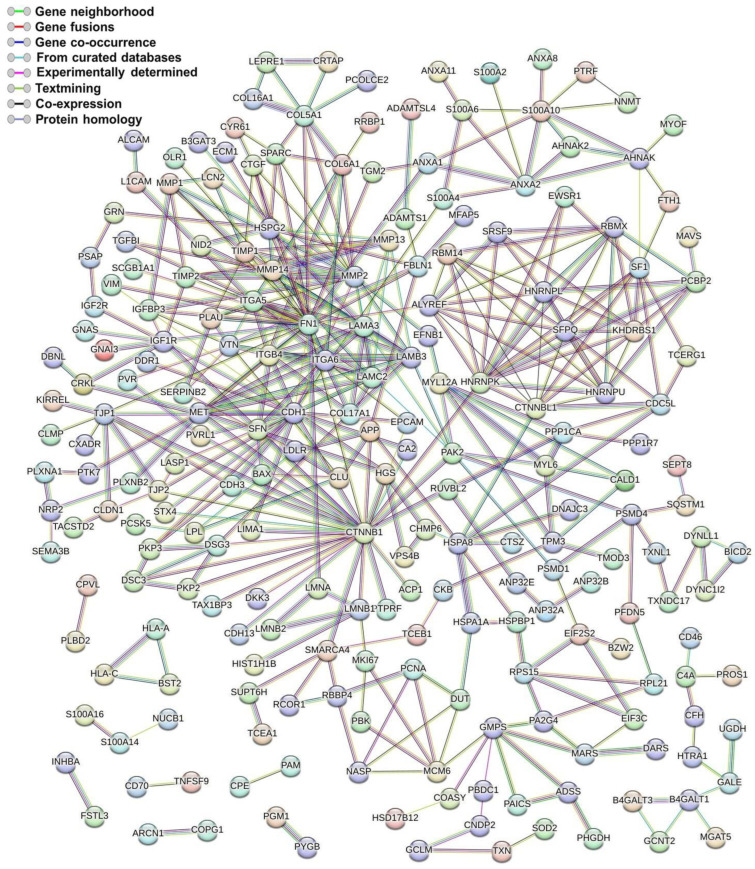
Protein–protein interaction (PPI) network of differentially expressed proteins from cervical cancer EVs. The network shows the 218 proteins that have at least 1 functional connection. The network nodes represent the proteins, and the edges represent the protein–protein associations. The line color connecting the nodes indicates the type of interaction, as shown in the top left corner.

A protein–protein interaction (PPI) network of the 322 differentially expressed proteins between cervical cancer cells and the control HaCaT cell line was constructed. These proteins formed an interaction network composed of 321 nodes and 422 edges. The network showed that 218 of the 322 proteins had at least 1 functional connection (Figure 6). The average node degree was 2.63, with an average local clustering coefficient of 0.386 and a PPI enrichment *p*-value < 1 × 10^−16^. This network had 422 interactions, significantly more than the 210 expected. This evidence indicates that the proteins identified in the EVs have more interactions between themselves than would be expected for a random set of proteins of the same size. This enrichment also indicates that the proteins are at least partially biologically connected as a group.

## 4. Discussion

Extracellular vesicles contain and transport diverse biomolecules and thus play an essential role in intercellular communication [20]. The EVs released by tumor cells may participate in the hallmarks of cancer via a horizontal transfer of functional biomolecules to cells in the TME [21,22]. An evaluation of the composition of the EVs produced by cervical cancer cells has mainly led to describing their RNA content [23,24,25,26], but the total protein content of these EVs remains unknown, as well as the proteins contained in these EVs with a possible function in this cancer. In this study, we analyzed the protein content of the EVs derived from cervical cancer using the HeLa cell line as a first step for detecting overexpressed molecules in the EVs from cervical cancer.

The proteins identified in the EVs derived from HeLa and HaCaT cell lines were compared with those of the Vesiclepedia database [13], and we identified 131 proteins not reported in this database, suggesting that they are particular to these types of EVs. The top 20 proteins reported in Vesiclepedia were also identified in our vesicles. We observed the main markers [27] such as PDCD6IP (ALIX), HSPA8 (HSP70), CD9, HSP90, and CD63.

Our analysis revealed evidence of differences in protein profiles between both EV groups, showing that the EVs from non-tumorigenic HaCaT keratinocytes contained more proteins than the EVs from HeLa cervical cancer cells. It has been reported that in some types of cancer, EV production is higher than in non-tumor cells [28,29,30]. However, it is unknown whether this ratio is proportional to the number of proteins they contain. We consistently observed similar quantities of total vesicular protein from the same number of initial keratinocytes.

As mentioned in the Introduction section, in cervical cancer EVs, only some proteins have been identified [6,17,18,19]. However, these reports do not show whether there is a difference in their expression levels compared with the EVs produced by non-tumorigenic keratinocytes. We identified most of the reported proteins, but we only found two significantly upregulated and one downregulated, which shows the need to identify not only the protein content that could still be present under physiological conditions but also those proteins overexpressed or downregulated in EVs under pathological conditions. If the up- and downregulated proteins in HeLa’s EVs are also modified in other cervical cancer cell lines and samples, they need to be evaluated to establish a signature or fingerprint of vesicular proteins in cervical cancer, but this is a first step.

We employed the EVs derived from non-tumorigenic HaCaT keratinocytes as the control group to determine the differentially expressed proteins; these types of keratinocytes are classically used as control keratinocytes due to the homogeneity offered for cell lines, their lack of finite lifespan, and near normal phenotype [31]. The number of proteins identified in the EVs of HaCaT cells was lower than that previously observed by Glady et al., who performed a proteomic analysis in which they identified 5143 proteins in the EVs of HaCaT cells; this led us to the conclusion that several HaCaT-exclusive proteins were not observed in our research. The differences between both results may be due to the method used to obtain EVs since they performed EV isolation via the affinity to TIM-4, a phospholipid present on the surface of EVs [32]. However, this methodology is not as widely employed for EV isolation as other methods such as ultracentrifugation or precipitation [33,34]. Therefore, the comparison of the results observed by Glady et al. and those surveyed in the EVs from other keratinocytes in different studies would not be adequate. The differences observed here in the protein content of the EVs from the HeLa cell line versus HaCaT could be attributable to the characteristics of the cancer cells as well as the viral oncogenes since HPV infection affect not only the protein synthesis in cells but also their loading into the EVs secreted. This last effect was observed by Honegger et al. by silencing the HPV 18 oncogenes E6 and E7 in the HeLa cell line, which modified both the protein content and the number of EVs released [17].

The identification of proteins from oncogenic viruses such as Epstein–Barr virus [35], hepatitis B virus [36], and hepatitis C virus [37] in EVs suggests that viruses could favor cancer progression through EVs [38]. Our results indicate the absence of HPV proteins in the EVs derived from HeLa cells, which agrees with the previously reported by Honegger et al., who showed the lack of E6 and E7 oncoproteins in the EVs derived from the HeLa cell line [17]. Nevertheless, the possibility of finding HPV proteins in the EVs from cervical cancer cells is not ruled out since in the EVs isolated from the CaSki cell line, the presence of the E6 oncoprotein, but not the E7 oncoprotein of HPV type 16, was reported [18]. This result may be due to the higher HPV copy number in the CaSki cell line and thus the higher production of E6 [39]. Additionally, HPV type 16 E6 and E7 oncoproteins have also been reported in the EVs derived from the head and neck cancer cell lines UPCI-SCC-90, UM-SCC-2, and UM-SCC-47, or the EVs from the serum of patients with oropharyngeal cancer [40,41].

In addition to several proteomic analyses of the cells from women with cervical cancer [42,43,44,45,46], Higareda-Almaraz et al. performed a proteomic analysis using 2D SDS–PAGE and MALDI-TOF of the cervical cancer cell lines HeLa, CaLo, SiHa, CaSki, ViBo, and C-33 A, with the immortalized keratinocyte cell line HaCaT as a control. They identified 66 differentially expressed proteins, termed the “central core of cervical cancer”. These proteins were present in all six cervical cancer lines and were more than two-fold changed or absent in HaCaT cells [43]; however, there are no reports about their presence or regulation in the EVs released from cervical cancer cells. Here, in the EVs of HeLa cells, we identified 63 of these 66 proteins, and we also observed the upregulation of VIM (vimentin) and CKB (creatine kinase B-type) in the EVs of HeLa, but further studies are required to assess whether an effect can be expected from VIM, CKB, and other upregulated proteins when receptor cells internalize these EVs. In the EVs from the C-33 A cell line, 59 of these “central core of cervical cancer” proteins were also identified. Although both cell lines are cervical cancer cell lines, the difference in the number of proteins identified in the EVs from HeLa and C-33 A (HPV negative) cells may be due to the influence of HPV oncoproteins, which modify protein packaging in EVs.

EVs are released into the extracellular milieu and, for this reason, are considered part of the cells’ secretome [47]. In cervical cancer cells, they have been evaluated from cultures of HeLa, SiHa, and C-33 A cell lines, as well as in the normal cervical keratinocyte HCK1T, using 2DE and MALDI-TOF-MS. Among the multiple proteins secreted by cervical cancer cells, four upregulated proteins were identified: nucleobindin-1 (NUCB1), carboxypeptidase E (CPE), calreticulin (CALR), and heat-shock protein beta-1 (HSPB1). Only the first three are classically secreted [48]; therefore, HSPB1 was the main protein expected to be in EVs. We identified all four proteins; NUCB1 and CPE were upregulated, and CALR was downregulated, but none with statistical significance, while HSPB1 showed no change between the two samples, which suggests their presence in EVs in physiological conditions. These proteins’ increase in the secretome may be attributable, besides an increased secretion of themselves, to the growth in the number of EVs, but this observation needs further evaluation. Additionally, multiple proteases were detected in the secretome of cervical cancer cells, such as ADAM10 (disintegrin and metalloproteinase domain-containing protein 10), CTSD (cathepsin D), and SOD2 (superoxide dismutase [Mn], mitochondrial), and some of them were dysregulated such as TIMP1 (metalloproteinase inhibitor 1) and TIMP2, which were reported to be upregulated, whereas MMP2 (matrix metalloproteinase-2) and MMP9 were not identified [49]. In comparison, we observed MMP2 and SOD2 to be downregulated in HeLa cell’s EVs and identified CTSD and ADAM10 but without any difference from the EVs from HaCaT cells.

Among other upregulated proteins, PRSS56 was the one with the highest FC. It is expressed in the HeLa cell line; however, its involvement in cervical cancer is unknown, and its function has been associated with regulating ocular growth [50]. STOM is another of the main upregulated proteins whose role in cervical cancer is unknown, but it has been proposed as a new exosomal marker after being identified in the EVs of breast, lung, and ovarian cancer cells, as well as in the EVs of body fluids such as blood plasma, uterine fluid, ascitic fluid, and gastric juice [51,52]. Among the upregulated proteins with the highest FC, we identified several proteins related to cervical cancer, such as MSLN (mesothelin), a cell surface glycoprotein linked to a role in cell adhesion. MSLN has been identified as upregulated in cervical cancer tissue samples and proposed as a tumor-associated antigen [53] or a target for cervical cancer therapy [54,55]. Even if its function is unclear, it has been attributed to a role in apoptosis since its silencing in SiHa cells promotes an increase in apoptosis without significant effects on proliferation and migration [55]. LPL also had one of the highest FC; it was upregulated in cervical squamous cell carcinoma, and their overexpression in the cervical cancer cell lines SW756 and C-33 A increased their invasiveness of them [56]. L1CAM is considered a prognostic marker in several types of cancer and has been suggested to be involved in epithelial–mesenchymal transition (EMT) [57]. Its presence in cervical cancer is associated with larger tumor size, shorter disease-free survival, and the expression of VIM (vimentin) [57,58]. HTRA1 (high-temperature requirement protein 1) has been considered a tumor suppressor. However, other studies have suggested that it may be a proto-oncogene [59]. In cervical cancer, the overexpression of HTRA1 in the CaSki cell line promoted cell proliferation without interfering with the rate of apoptosis [60].

The BPs with the highest number of up- and downregulated proteins were cell adhesion, proteolysis, and extracellular matrix (ECM) organization. Several proteins related to these processes have been previously described in the EVs of different types of cancer from various fluids. Some of these proteins include ALCAM, basigin, CD44, EpCAM (epithelial cell adhesion molecule), integrin alpha-5, integrin beta-1, L1CAM, and MCAM (cell surface glycoprotein MUC18) [61], integrin alpha-3, integrin beta-8, ephrin-B2, ephrin receptors (EphA2, EphB1, and EphB4), thrombospondin-1, junctional adhesion molecule A, claudin-3, protocadherin-1 [62], various ADAMs, soluble ADAM whit thrombospondin motif (ADAMTS), and other MMPs as well as their endogenous inhibitors (TIMPs) [63,64]. Around 11 MMPs, 6 ADAMs, 7 ADAMTS, and 3 TIMPs have been reported in EVs of various origins [63]. Some of these proteins are included in the mentioned BPs, and therefore EVs may be involved in processes associated with tumor development such as migration, invasion, angiogenesis, and metastasis, but this needs to be evaluated, as well as the protein involved in EVs.

Our analysis of the biological pathways revealed that the immune response proteins were deregulated in the EVs from HeLa. In other cells, it has been observed that some proteins such as TGF-β, ICOSL, TRAIL, PD-L1, and FASL could participate in evading the immune response when they are transferred through EVs [65]. However, to date, no proteins have been described in cervical cancer’s EVs that participate in immune response pathways, and there are few reports about the role or the effects of cervical cancer’s EVs in immune cells. Iuliano et al. showed that the E6 and E7 oncoproteins of HPV 16 and HPV 38 affect the packaging of immune system molecules into EVs since the transduction of E6/E7 oncogenes in primary human foreskin keratinocytes altered the mRNA expression levels of the cytokines and chemokines contained in the EVs derived from these cells [66]. The MVs released from HaCaT cells transduced with the E7 oncogene also affect immune system cells. Co-culture of Langerhans cells (LCs) with these MVs decreased the cytotoxicity of CD8+ T lymphocytes induced by them. This observed effect suggests that the MVs released by HPV (+) cells may inhibit the T lymphocyte response by modulating LCs and thus contribute to the persistence of infection and cancer development [67]. However, Ren et al. found opposite results with HeLa’s EVs; dendritic cells loaded with the exosomes derived from the HeLa cell line stimulated proliferation and cytotoxicity of CD8+ T lymphocytes; subsequently, these inhibited the growth of HeLa cells, thus demonstrating increased cytotoxicity and an antitumor effect [68]. More analysis is needed to confirm whether the observed effects are due to the different protein content of these EVs, which we observed in our research.

Today, less than one hundred original reports exist to characterize cervical cancer EVs, and some effects on their receptor cells have been mainly evaluated with cell lines. However, the observed results from the EVs of cervical cancer and other different malignancies propose them as potential therapeutic or diagnostic targets for this cancer. Initially, their utility as biomarkers was suggested due to the detection of their protein content (KPNB1, CRM1, CAS, IPO5, and TNPO1) in the serum of patients with cervical cancer [69]. However, the evaluation of any EV molecule as a possible marker or therapeutic target will be random if it is unknown whether it is found to be modified in the EVs in this cancer. We made a first step, but more efforts are necessary to prove if up- and downregulated proteins are included in the EVs of keratinocytes isolated from samples with cancer, along with their effect on receptor cells.

## 5. Conclusions

The results of our proteomic analysis indicate that the EVs released by cervical cancer cells contain upregulated proteins described for their involvement in this type of cancer. Moreover, the content of proteins related to the processes of cell adhesion, proteolysis, and extracellular matrix organization in these EVs could favor the migration, invasion, and metastasis of tumor cells. Finally, several upregulated proteins of these EVs could have an essential role due to their participation in the biological pathways associated with the immune system, so we can infer that EVs probably play a crucial role in activating or suppressing immune system cells in cancer.

## Figures and Tables

**Table 1:** Top twenty most reported proteins in EVs according to the Vesiclepedia database and found using LC-MS/MS in HeLa and HaCaT EVs.

Position in the Top 100	Gene Symbol	Protein Name	Average LFQ HeLa’s EVs	Average LFQ HaCaT’s EVs
1	*PDCD6IP*	Programmed cell death 6 interacting protein	30.90126	31.82523
2	*GAPDH*	Glyceraldehyde-3-phosphate dehydrogenase	34.12177	34.66822
3	*HSPA8*	Heat-shock 70kDa protein 8	33.61965	34.98863
4	*ACTB*	Actin, cytoplasmic 1	36.35891	36.49115
5	*ANXA2*	Annexin A2	32.45719	35.18595
6	*CD9*	CD9 antigen	30.40349	30.71279
7	*PKM*	Pyruvate kinase PKM	33.28324	34.29517
8	*HSP90AA1*	Heat-shock protein HSP 90-alpha	34.42612	34.92483
9	*ENO1*	Alpha-enolase	33.79492	33.82500
10	*ANXA5*	Annexin A5	31.05360	31.50710
11	*HSP90AB1*	Heat-shock protein HSP 90-beta	33.63702	33.91836
12	*CD63*	CD63 antigen	29.42370	29.26211
13	*YWHAZ*	14-3-3 Protein zeta/delta	31.30615	32.74208
14	*YWHAE*	14-3-3 Protein epsilon	30.73296	31.84702
15	*EEF1A1*	Elongation factor 1 alpha 1	32.55905	33.68600
16	*PGK1*	Phosphoglycerate kinase 1	31.74001	31.45923
17	*CLTC*	Clathrin heavy chain 1	26.95375	28.79626
18	*PPIA*	Peptidyl-prolyl cis-trans isomerase A	31.76701	32.57919
19	*SDCBP*	Syntenin-1	30.96517	32.68455
20	*ALDOA*	Fructose-bisphosphate aldolase A	32.53601	33.17732

## Data Availability

Not applicable.

## Supplementary Materials

The following supporting information can be downloaded at: https://www.mdpi.com/article/10.3390/v15030702/s1, Figure S1: Identification of HPV in the HeLa cell line via PCR; Figure S2: Heat map of the 322 differentially expressed proteins in EVs from HeLa and HaCaT cell lines; Figure S3: Validation of differentially expressed proteins using Western blot; Table S1: Identified proteins; Table S2: Analysis in Vesiclepedia; Table S3: Top 100 proteins Vesiclepedia; Table S4: Up- and downregulated proteins; Table S5: Identification via qualitative proteomic analysis; Table S6: Gene ontology of up- and downregulated proteins; Table S7: Reactome biological pathways.
