# Supplementary material for: Quantitative Proteomics for the Identification of Differentially Expressed Proteins in the Extracellular Vesicles of Cervical Cancer Cells
Source: Viruses. 2023 Mar 8;15(3):702. doi: 10.3390/v15030702 (PMC10051161; doi:10.3390/v15030702)
Supplement: Supplementary file 1 [file viruses-15-00702-s001.zip › Supplementary Figure1.pdf]

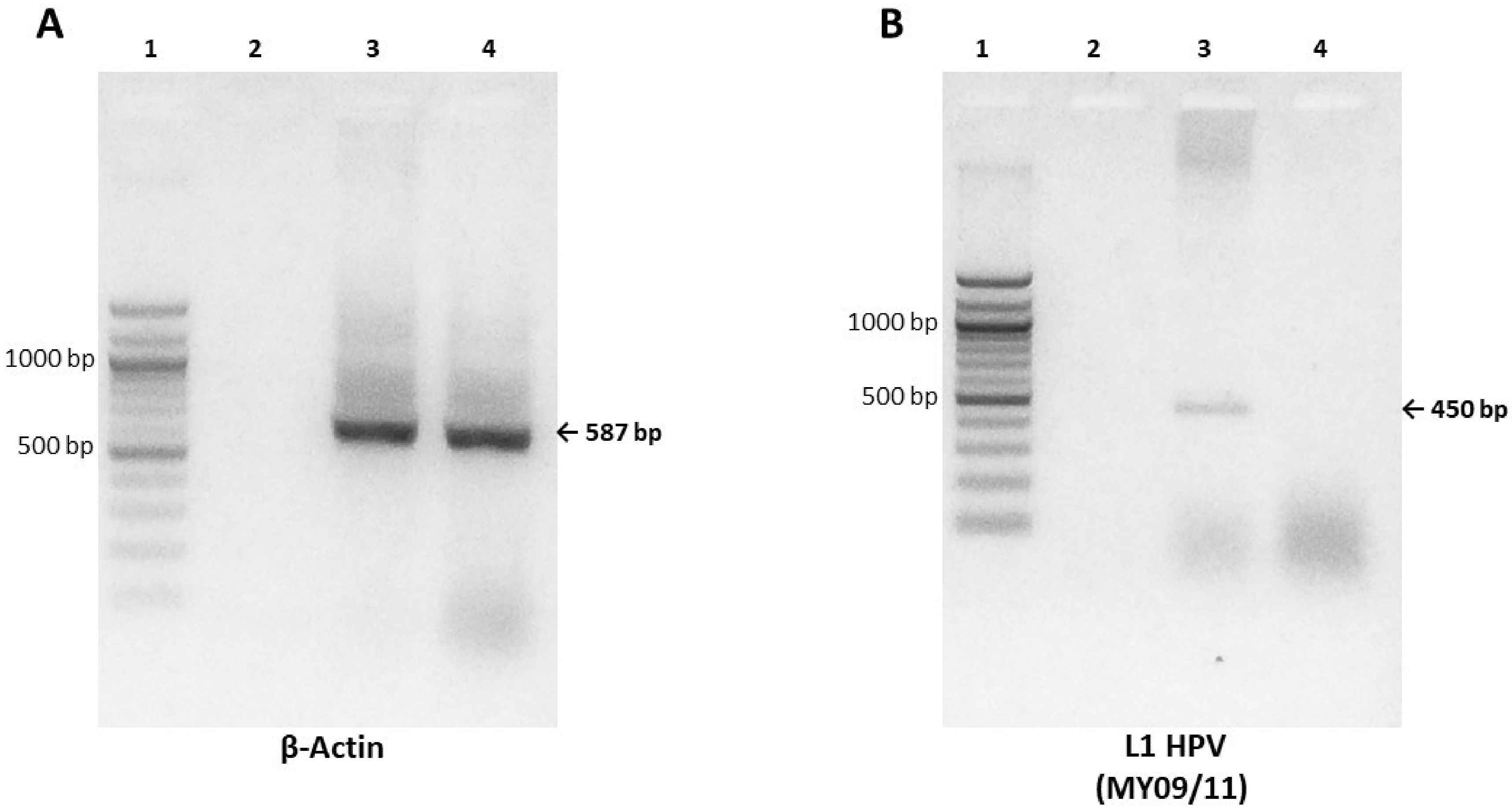

Figure S1. Identification of HPV in the HeLa cell line by PCR. DNA from HeLa and HaCaT cell lines was isolated and amplified by PCR; amplification products were visualized on 2% agarose gels. A) Amplification of a 587 bp product of the human *Beta-actin* housekeeping gene. B) 450 bp amplicon of the L1 region of HPV with primers MY09/11. (1) Molecular weight ladder; (2) Negative control; (3) HeLa cells; (4) HaCaT cells.
