# Supplementary material for: Quantitative Proteomics for the Identification of Differentially Expressed Proteins in the Extracellular Vesicles of Cervical Cancer Cells
Source: Viruses. 2023 Mar 8;15(3):702. doi: 10.3390/v15030702 (PMC10051161; doi:10.3390/v15030702)

Figure S2. Heat map of the 322 differentially expressed proteins in EVs from HeLa and HaCaT cell lines. Up-regulated proteins are shown in red, while down-regulated proteins are shown in green. HeLa01 and HeLa02 correspond to the two biological replicates of the cervical cancer EVs group; HaCaT01 and HaCaT02 correspond to the two biological replicates of the control group.

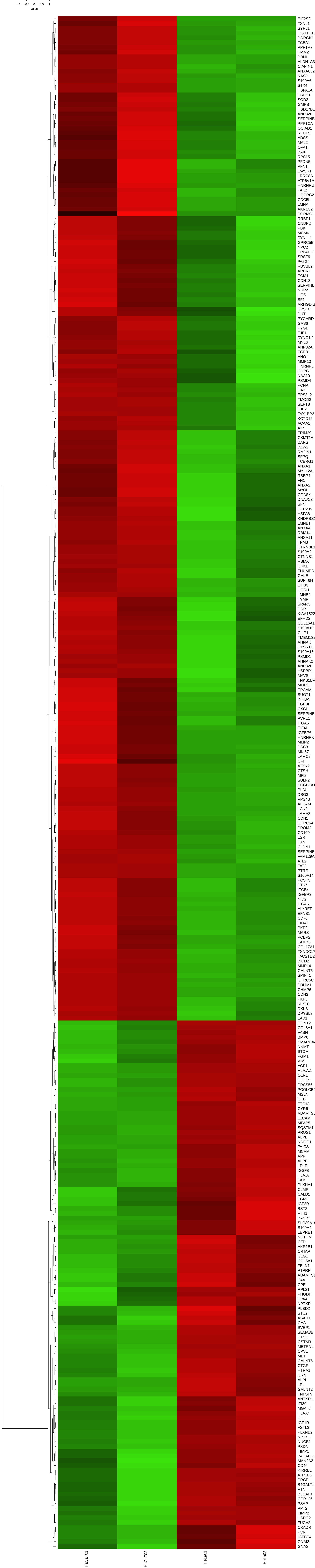

Supplement: Supplementary file 1 [file viruses-15-00702-s001.zip › Supplementary Figure2.pdf]
