# Supplementary material for: Quantitative Proteomics for the Identification of Differentially Expressed Proteins in the Extracellular Vesicles of Cervical Cancer Cells
Source: Viruses. 2023 Mar 8;15(3):702. doi: 10.3390/v15030702 (PMC10051161; doi:10.3390/v15030702)
Supplement: Supplementary file 1 [file viruses-15-00702-s001.zip › Supplementary Figure3.pdf]

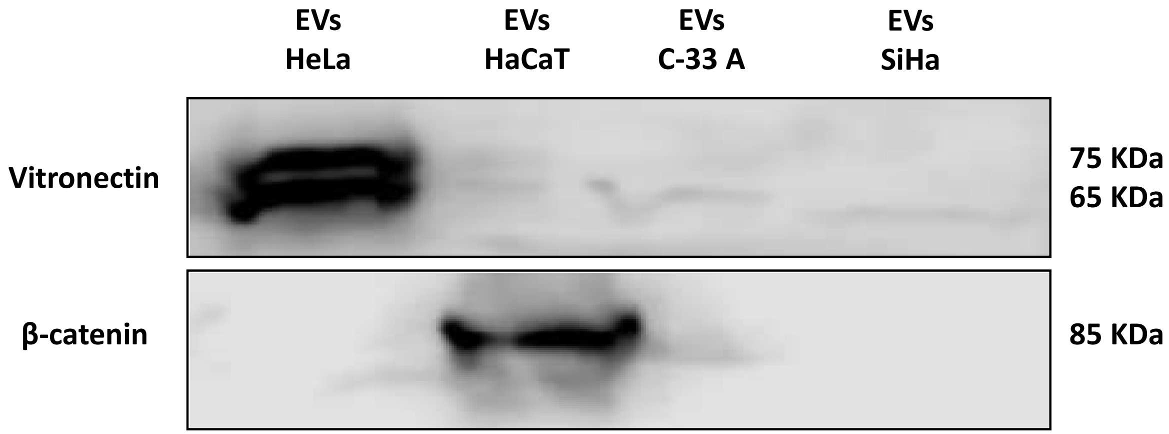

Figure S3. Validation of differentially expressed proteins by western blot. Vitronectin (up-regulated) and β-catenin (down-regulated) proteins evaluated in EVs from HeLa, C-33 A and SiHa cervical cancer cell lines, in addition to EVs from control HaCaT keratinocytes.
